# Magnetite Nanoparticles In-Situ Grown and Clustered on Reduced Graphene Oxide for Supercapacitor Electrodes

**DOI:** 10.3390/ma15155371

**Published:** 2022-08-04

**Authors:** Yue Jiang, Jinxun Han, Xiaoqin Wei, Hanzhuo Zhang, Zhihui Zhang, Luquan Ren

**Affiliations:** 1 Key Laboratory of Bionic Engineering of Ministry of Education, College of Biological and Agricultural Engineering, Jilin University, Changchun 130022, China; 2 School of Materials and Physics, China University of Mining & Technology, Xuzhou 221116, China

**Keywords:** magnetite, supercapacitors, graphene, coprecipitation, electrochemistry

## Abstract

Fe_3_O_4_ nanoparticles with average sizes of 3–8 nm were in-situ grown and self-assembled as homogeneous clusters on reduced graphene oxide (RGO) via coprecipitation with some additives, where RGO sheets were expanded from restacking and an increased surface area was obtained. The crystallization, purity and growth evolution of as-prepared Fe_3_O_4_/RGO nanocomposites were examined and discussed. Supercapacitor performance was investigated in a series of electrochemical tests and compared with pure Fe_3_O_4_. In 1 M KOH electrolyte, a high specific capacitance of 317.4 F g^−1^ at current density of 0.5 A g^−1^ was achieved, with the cycling stability remaining at 86.9% after 5500 cycles. The improved electrochemical properties of Fe_3_O_4_/RGO nanocomposites can be attributed to high electron transport, increased interfaces and positive synergistic effects between Fe_3_O_4_ and RGO.

## 1. Introduction

Since the last two decades, various energy devices, such as batteries, fuel cells and supercapacitors, have been developed in order to alleviate the energy crisis and environmental problems [1,2,3]. Among them, pseudocapacitors have intrigued much attention due to their exceptional advantages, including higher power density, high rates of charge-discharge, cost reduction and relative safety [4]. Transition metal oxides are considered as ideal electrode materials for pseudocapacitors because of their high theoretical specific capacitance and high electrochemical activity. RuO_2_ has been revealed as the first pseudocapacitive electrode material with an excellent pseudocapacitance of about 720 F g^−1^ [5]. However, its prohibitive cost and toxic nature has motivated the search for economical and environment-friendly alternatives with equivalent performance. Other transition metal oxides, such as Co_3_O_4_, NiO, MnO_2_ and Fe_3_O_4_, have been studied extensively as substitutes for Ru-based oxide electrodes [6,7,8,9]. Among them, Fe_3_O_4_ has been identified as the promising material, considering its high theoretical specific capacitance (2299 F g^−1^), low cost and large potential window. In an early study, Fe_3_O_4_ hollow spheres possessed a capacity of 294 F g^−1^ and 90.8% capacity retention after 500 cycles [9]. However, research on Fe_3_O_4_ as electrodes have shown relatively low capacitances below 400 F g^−1^ [9,10,11]. The reason might be related to its limited electrical conductivity that hinders ion diffusions. So far, exploring novel Fe_3_O_4_-based materials for supercapacitors application is still challenging.

Nanostructured Fe_3_O_4_ composited with carbonaceous materials has been reported as an effective strategy that may offer higher energy density with larger specific capacitance. For instance, Fe_3_O_4_ composited with carbon nanotubes, exhibited improved cyclic stability and energy density in contrast to those of pure Fe_3_O_4_ [12]. The specific capacity of 275.9 F g^−1^ was acquired at 0.5 A g^−1^ in Na_2_SO_4_ aqueous solution for carbon-coated Fe_3_O_4_ composites [13]. Recently, reduced graphene oxide (RGO) has attracted much attention on account of its tremendous electronic conductivity and high surface area. Considerable efforts have been made to design and synthesize Fe_3_O_4_/RGO composites for supercapacitor applications. Kumar et al. prepared 3D network of Fe_3_O_4_/RGO composites by one-pot microwave approach and acquired specific capacity of 297.0 F g^−1^ at 4.4 A g^−1^ [14]. Another piece of research on Fe_3_O_4_ nanorods decorated on RGO surfaces in two-step procedures achieved 95% specific capacity retention after 2000 cycles [15]. The improved electrochemical performance with the presence of RGO is found to be prevalent and strongly dependent on the synergistic effects between Fe_3_O_4_ and RGO [15]. Therefore, it is highly desirable to design Fe_3_O_4_/RGO composites with combined interfaces for specific capacitance enhancement.

In this paper, Fe_3_O_4_/RGO nanocomposites with unique microstructures were prepared by a facile coprecipitation process. By introducing certain additives, Fe_3_O_4_ nanoparticles could be anchored on RGO surfaces and formed as nanoclusters without aggregation. The interlayer spacing between RGO sheets was expanded by these Fe_3_O_4_ nanoclusters, which contributed to ion transport in the electrolyte. In a series of electrochemical tests, the as-prepared Fe_3_O_4_/RGO nanocomposites exhibited high specific capacitance, good rate capability and long cyclic stability.

## 2. Materials and Methods

### 2.1. Preparation of Fe_3_O_4_/RGO Nanocomposites

In a typical procedure, Fe_3_O_4_/RGO nanocomposites were synthesized as follows: First, 0.20 g GO was dispersed into 200 mL distilled water and ultrasonicated for 5 h to form a homogeneous suspension. Then, it was put into a five-neck flask with a thermostatic water bath around 80 °C. Second, 5.56 g FeSO_4_·7H_2_O was dissolved and added into the flask with constant stirring while Ar was forced into the mixed solution to obtain an oxygen-free environment. Subsequently, 14.47 g NH_4_Fe(SO_4_)_2_·12H_2_O and 20 mL polyethylene glycol (PEG-400) were dissolved, respectively, and added into the flask successively. Third, 4.80 g NaOH dissolved in 100 mL distilled water was dropwised into the flask for 1 h, while the resultant solution was maintained for another 1 h to complete the coprecipitation. Corresponding reactions can be described as:

Fe^2+^ + 2NaOH = Fe(OH)_2_↓+ 2Na^+^ (1)

Fe^3+^ + 3NaOH = Fe(OH)_3_↓+ 3Na^+^ (2)

Fe(OH)_2_ + 2Fe(OH)_3_ = Fe_3_O_4_ + 4H_2_O (3)

**Figure 1:**
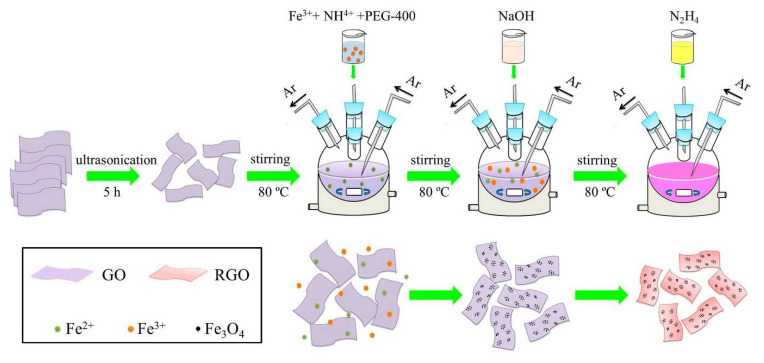
Schematic illustration of the synthesis process and growth evolution of Fe_3_O_4_/RGO nanocomposites.

Finally, 4 mL N_2_H_4_·H_2_O (85%) was dropwised into the flask for 1 h. Then, the reaction system was kept for 3 h to accomplish the reduction from GO to RGO. After the reaction was completed, the black products were washed several times, then dried at 50 °C for 9 h. According to the above synthesis procedures, the molar ratio of Fe_3_O_4_ to RGO in the as-prepared composites was estimated to be 2:1. The schematic for synthesizing Fe_3_O_4_/RGO nanocomposites was illustrated in Figure 1. For comparison, Fe_3_O_4_ nanoparticles were prepared under the similar experimental condition where GO was removed from the flask.

### 2.2. Material Characterization

The as-prepared GO, Fe_3_O_4_ nanoparticles, and Fe_3_O_4_/RGO nanocomposites were examined by X-ray diffraction (XRD, Rigaku D/max 2500PC, Tokyo, Japan) and Fourier transform infrared (FT-IR, Bruker Tensor 27, Billerica, MA, USA) spectroscopy, respectively. Microstructure and morphology analysis were conducted on transmission electron microscope and high-resolution transmission electron microscopy (TEM & HRTEM, FEI TecnaiG^2^F20, Lincoln, NE, USA). To quantify the chemical compositions, X-ray photoelectron spectroscopy (XPS, Thermo Fisher ESCALAB 250Xi, Waltham, MA, USA) measurements were performed by using 300 W Al Kα radiations as the X-ray source for excitation.

### 2.3. Electrochemical Measurements

The working electrodes were prepared by mixing 80 wt.% active material, 10 wt.% acetylene black and 10 wt.% polyvinylidene fluoride dissolved to form a slurry and coated on nickel foam (10 mm × 15 mm). The electrochemical investigations were employed in 1 M KOH electrolyte. Platinum and Hg/HgO were used as counter and reference electrodes, respectively. Electrochemical performances were detected by Chi660e electrochemical workstation.

## 3. Results and Discussion

**Figure 2:**
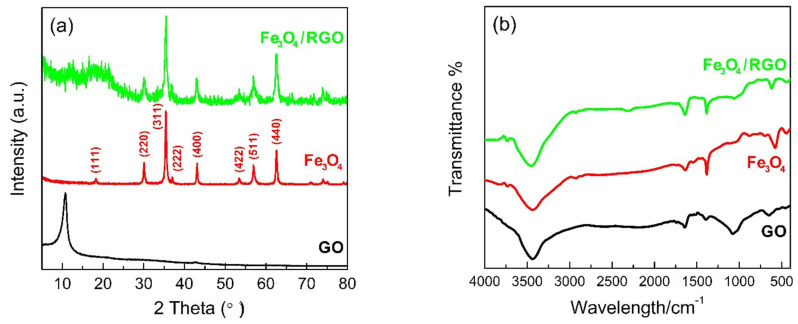
(**a**) XRD patterns and (**b**) FT-IR spectrum of as-prepared GO, Fe_3_O_4_ nanoparticles and Fe_3_O_4_/RGO nanocomposites.

XRD results of as-prepared GO, Fe3O4 nanoparticles and Fe3O4/RGO nanocomposites are shown in Figure 2a. The as-prepared GO presents a strong diffraction peak around 2θ = 10.5°, corresponding to its characteristic (001) plane. The layer-to-layer distance of GO is calculated to be 0.83 nm by Scherrer equation, which is much larger than that of graphite. The introduction of the oxygen-containing functional groups between GO sheets should be responsible for it [14]. For Fe3O4 nanoparticles, their sharp and strong diffraction peaks are indexed to spinel phase (JCPDS: No.65-3107), indicating a preferable crystallization and high purity. The diffraction peak positions of Fe3O4/RGO nanocomposites are well-matched with Fe3O4 nanoparticle, where the decreased peak intensities and relatively broadened peaks suggest their reduced crystallinity [16]. FT-IR analysis results of the as-prepared samples were collected by attenuated total reflection method and preprocessed by OMNIC software (version number 9.2). As is shown in Figure 2b, distinct peaks around 3430 cm^−1^ are prevalent in all spectra, which is related to the stretching vibrations of O-H bands. The other three characteristic peaks in the spectrum of GO should be ascribed to C=C (1643 cm^−1^), CO-H (1388 cm^−1^) and C-O (1082 cm^−1^) stretching vibrations [17]. In the spectra of Fe3O4 nanoparticles and Fe3O4/RGO nanocomposites, stretching vibrations of Fe-O bands around 580 cm^−1^ are conspicuous [15]. For Fe3O4/RGO nanocomposites, another peak around 1646 cm^−1^ is accurate and confirmed the existence of RGO. Compared with GO, the spectrum of Fe3O4/RGO has no infrared peaks around 1082 cm^−1^, implying that GO has been completely reduced into RGO after hydrothermal processes.

**Figure 3:**
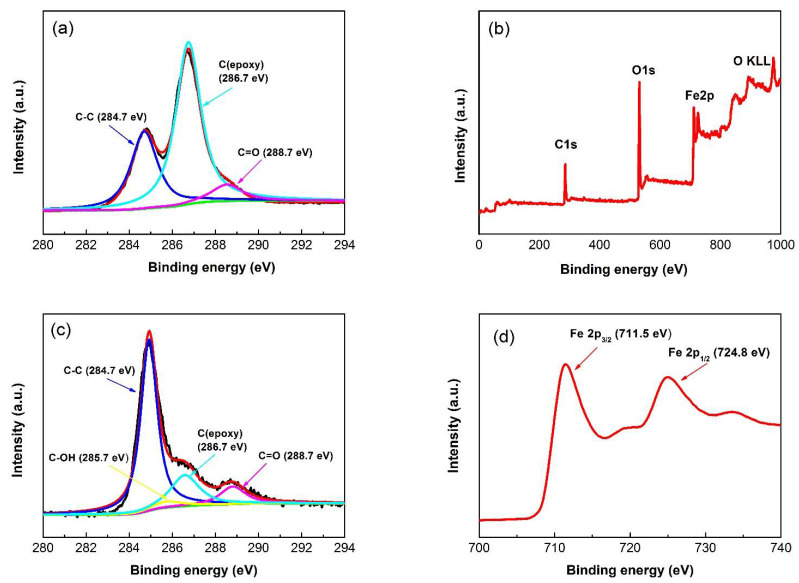
XPS spectrum of (**a**) C 1s for as-prepared GO, (**b**) wide scanning for Fe_3_O_4_/RGO nanocomposites with (**c**) C 1s and (**d**) Fe 2p spectral profiles.

Chemical compositions and oxidation states of as-prepared GO and Fe_3_O_4_/RGO nanocomposites were determined by XPS analysis. C 1s spectrum of GO in Figure 3a can be deconvoluted into three different peaks. The peaks centered at 284.7 eV, 286.7 eV and 288.7 eV should be indexed to C–C/C=C, C–O and C=O groups, respectively [18]. The survey spectrum of Fe_3_O_4_/RGO nanocomposites in Figure 3b confirms the existence of Fe, C, and O elements, while no other elemental signals can be detected. Compared with Figure 3a, the peak of sp^2^ hybridized C–C /C=C bonds is increased, while the peaks of both C–O and C=O bonds are significantly decreased in Figure 3c, implying the adequate reduction from GO to RGO. For Fe 2p spectrum in Figure 3d, two major peaks at 711.5 eV and 724.7 eV were identified as Fe 2p_3/2_ and Fe 2p_1/2_, respectively, demonstrating Fe^2+^ and Fe^3+^ ions in Fe_3_O_4_ [16].

**Figure 4:**
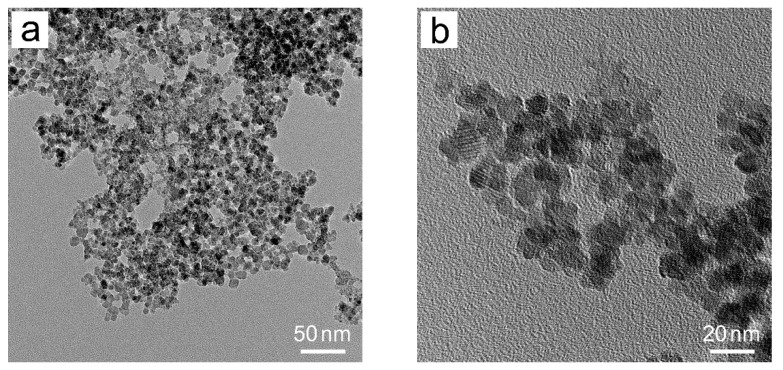
TEM images in (**a**) low and (**b**) high magnifications of Fe_3_O_4_ nanoparticles.

**Figure 5:**
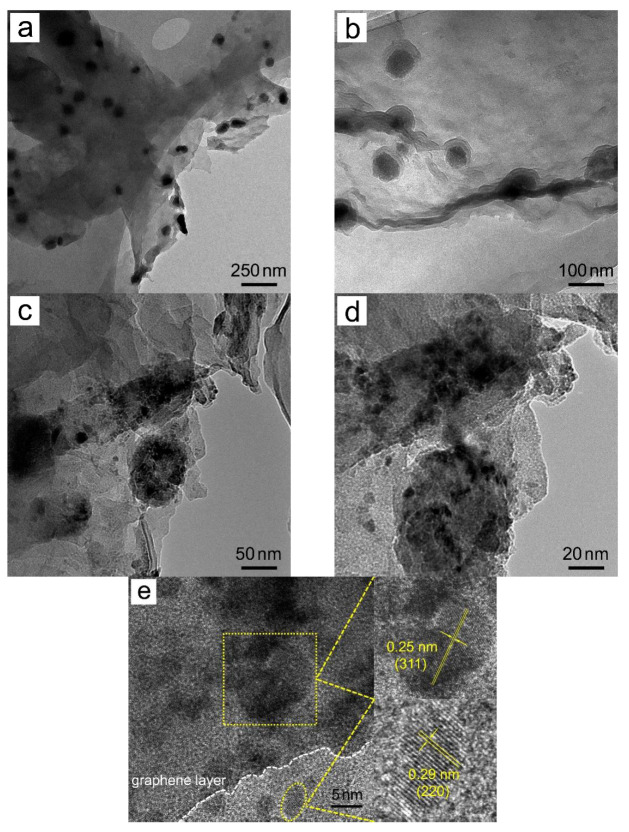
(**a**–**d**) TEM and (**e**) HRTEM images of Fe_3_O_4_/RGO nanocomposites where the selected areas in (**e**) are enlarged and shown in the top and bottom right corners, respectively.

Figure 4 displays the TEM images of Fe_3_O_4_ nanoparticles prepared without the addition of GO. All the particles are approximately spherical with uniform sizes around 10 nm while agglomeration among these nanoparticles is inevitable. Similar experimental results have been reported in previous studies [18]. The TEM & HRTEM images of Fe_3_O_4_/RGO nanocomposites are given in Figure 5. From relatively low magnification, it can be seen that Fe_3_O_4_ nanoclusters with irregular ellipsoid shapes are dispersed uniformly and wrapped by RGO sheets, where the sizes of nanoclusters are in range of 45–110 nm. Accordingly, the interlayer spacing between RGO sheets is expanded by these magnetite nanoclusters, which avoids RGO from restacking. Figure 5c,d indicate that each Fe_3_O_4_ nanocluster is composed of dozens of tiny Fe_3_O_4_ nanoparticles without agglomeration. These self-assembled nanoparticles are anchored on RGO sheets with sizes around 3–8 nm, and some foldings can be observed at the edges of RGO sheets, which increase the surface area. Figure 5e illustrates the distinct interfaces between Fe_3_O_4_ nanoparticles and RGO sheets. HRTEM lattice fringes with d-spacing distances of 0.25 nm and 0.29 nm were determined from the top and bottom right corners of Figure 5e, corresponding to (311) and (220) planes of Fe_3_O_4_, respectively. Some RGO sheets are overlapped and corrugated due to the embedded and stabilized Fe_3_O_4_ nanoclusters on RGO support. Such unique microstructures could hinder the agglomeration of Fe_3_O_4_ nanoparticles and facilitate ion transport inside RGO sheets. Therefore, improved electrochemical performance of Fe_3_O_4_/RGO nanocomposites is anticipated in the following studies.

The growth evolution of Fe_3_O_4_/RGO nanocomposites is based on the synthesis processes with the effects of additives. GO sheets achieved from the expanded graphite generally contain abundant hydroxyl, epoxy, carboxyl and carbonyl functional groups [18]. Fe^3+^ and Fe^2+^ ions added into GO suspension tend to be attached by these functional groups owing to electrostatic attraction, which might be served as nucleation centers in coprecipitation reactions. The introduction of NH^4+^ and PEG-400 makes Fe_3_O_4_ nucleus grow selectively and inhibits agglomeration [19,20]. After the reduction from GO to RGO by hydrazine hydrate, Fe_3_O_4_/RGO nanocomposites with unique microstructures could be obtained. Compared with hydrothermal [21] and sol–gel routes [22], coprecipitation processes in this study have several advantages, including high yield, simple equipment, relative low temperature and efficiency.

**Figure 6:**
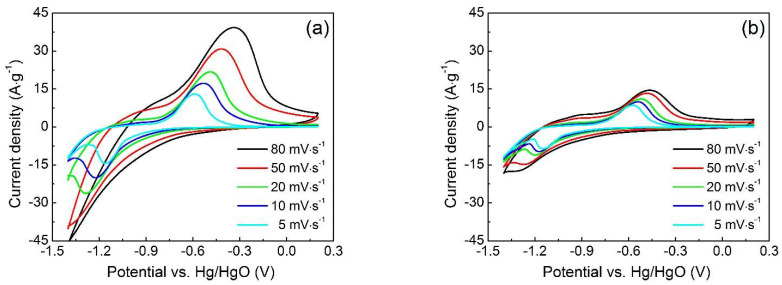
CV curves of (**a**) Fe_3_O_4_/RGO nanocomposites and (**b**) Fe_3_O_4_ nanoparticles at various scan rates in 1 M KOH solution.

Figure 6a illustrates the cyclic voltammetry (CV) curves of Fe_3_O_4_/RGO nanocomposites at various scan speeds. A lack of symmetry in the pairs of cathodic and anodic peaks is evident, which demonstrates pseudocapacitance. These redox peaks are assigned to reversible faradic reactions between Fe^2+^ and Fe^3+^ [23]. By increasing scan rates, distance between oxidation peak and reduction peak increases, which is due to the resistance of electrode. Additionally, the well-preserved shapes of CV curves at various scan rates imply the efficient electrochemical transportation and reversibility [24]. For comparison, the CV curves of Fe_3_O_4_ nanoparticles (Figure 6b) indicate much lower specific capacitance than that of the Fe_3_O_4_/RGO nanocomposites. The specific capacitance (*C_s_*) of Fe_3_O_4_ nanoparticles and Fe_3_O_4_/RGO nanocomposites can be calculated by the following equation:

(4) $$C_{s}=\frac{1}{sm\left(V_{a}-V_{b}\right)}\int_{V_{b}}^{V_{a}}idV$$

where *s* is scan rate, *m* is mass of electrode materials, *i* is respond current density, $V_{a}$ and $V_{b}$ are the integration limits of the voltammetric curves, respectively. At the scanning speed of 5 mV s^−1^, the calculated specific capacitance of Fe_3_O_4_/RGO nanocomposites and Fe_3_O_4_ nanoparticles are 572.8 F g^−1^ and 418.3 F g^−1^, respectively. The higher specific capacitance of Fe_3_O_4_/RGO nanocomposites is attributed to the existence of RGO providing high electrical conductivity and inhibiting the aggregation of Fe_3_O_4_ nanoparticles [25].

**Figure 7:**
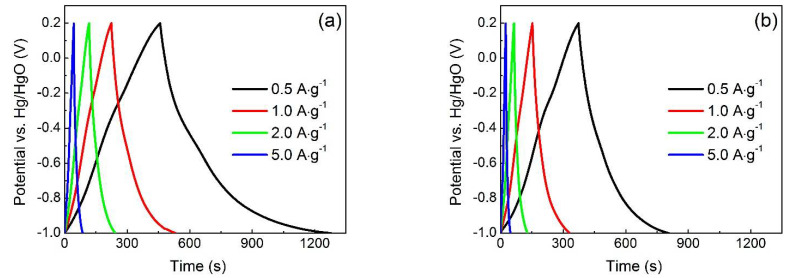
GCD curves of (**a**) Fe_3_O_4_/RGO nanocomposites and (**b**) Fe_3_O_4_ nanoparticles at various discharge current densities in 1 M KOH solution.

Typical galvanostatic charge–discharge (GCD) curves of Fe_3_O_4_/RGO nanocomposites and Fe_3_O_4_ nanoparticles are shown in Figure 7a,b, respectively. The charge and discharge curves of both electrode materials are approximately symmetrical in shape with a slight curvature, indicating a pseudocapacitor characteristic [26]. In contrast to Fe_3_O_4_ nanoparticles, Fe_3_O_4_/RGO nanocomposites have much longer discharge times at the same current density, which illustrates improved capacitive performance. The specific capacity can also be calculated by following equation: *C′_s_* = *I*Δt/*m*Δv, where *I*, Δt and Δv referred to discharge current, discharge time and voltage window, respectively. *C′_s_* value of Fe_3_O_4_/RGO nanocomposites is calculated to be 317.4 F g^−1^ at scan rate of 0.5 A g^−1^, which is almost twice that of Fe_3_O_4_ nanoparticles (169.1 F g^−1^). The specific capacitances of Fe_3_O_4_/RGO nanocomposites are 264.5, 223.7 and 198.1 F g^−1^, respectively, at current densities of 1.0, 2.0 and 5.0 A g^−1^. Even with a 10-fold increase in current density, 62.4% of the initial specific capacitance can still be maintained, showing the good rate performance of Fe_3_O_4_/RGO nanocomposites.

**Figure 8:**
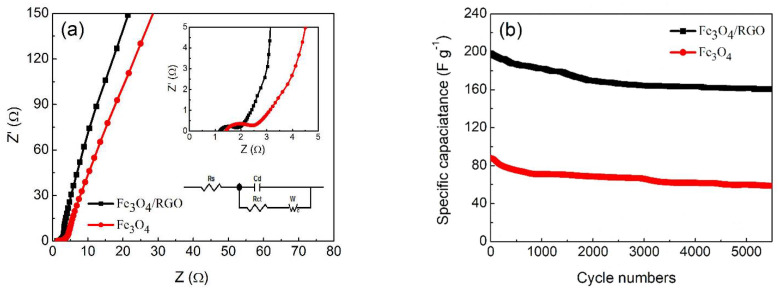
(**a**) Nyquist impedance plots of Fe_3_O_4_ nanoparticles and Fe_3_O_4_/RGO nanocomposites with insets of high-frequency regions and matched circuit diagram, (**b**) Cyclic performance of Fe_3_O_4_ nanoparticles and Fe_3_O_4_/RGO nanocomposites in 1 M KOH solution at a current density of 5.0 A g^−1^.

The charge transport and ionic diffusion characteristics of Fe_3_O_4_/RGO nanocomposites and Fe_3_O_4_ nanoparticles were determined by electrochemical impedance spectroscopy (EIS) measurements. As shown in Figure 8a, Nyquist plots of both electrode materials include semicircle parts at higher frequencies and straight-lined parts in lower frequencies. Generally, the semicircle parts represent charge transfer resistance (R_ct_) while the straight-lined parts attribute to Warburg impedance [27]. Corresponding circuit diagrams were matched with the help of Zview software and given in the inset of Figure 8a. Fe_3_O_4_/RGO nanocomposites exhibit a smaller semicircle in higher frequency ranges due to the existence of RGO [28]. Accordingly, R_ct_ values for Fe_3_O_4_/RGO nanocomposites is around 1.45 Ω, much lower than that of Fe_3_O_4_ particles (2.57 Ω), demonstrating their preferable electron transference. The straight line at lower frequencies close to 90º indicates ideal capacitive behavior. The oblique line of the Fe_3_O_4_/RGO nanocomposites is more vertical than Fe_3_O_4_ particles, revealing more efficient access of electrolyte ions. The intrinsic resistance (R_s_) of Fe_3_O_4_/RGO nanocomposites is about 1.18 Ω, lower than that of the Fe_3_O_4_ particles (1.41 Ω), which confirms the improved ion transport [28]. The cycling stability of both Fe_3_O_4_/GO nanocomposites and Fe_3_O_4_ nanoparticles were examined at the current density of 5.0 A g^−1^ for 5500 cycles, and the results were presented in Figure 8b. It can be figured out that the capacitance of both materials drops rapidly within 1000 cycles, then for Fe_3_O_4_ nanoparticles it maintains about 69.2% of their initial capacitance after 5500 cycles, while the capacitance retention of Fe_3_O_4_/RGO nanocomposite is as high as 86.9%.

Table 1 lists the electrochemical performance of Fe_3_O_4_ and related carbonaceous composites as supercapacitor electrodes in the present study and literature survey. It can be seen that pure Fe_3_O_4_ [9,10,11] has limited specific capacitance and cycle stability than its carbonaceous composites, owing to its limited electrical conductivity and agglomeration characteristics. Moreover, Fe_3_O_4_ composited with RGO [14,15,21,26] generally exhibits better performance, since RGO possesses a larger specific surface area than other carbonaceous forms [12,13]. Considering the complicated procedures to synthesize samples in the literature, the as-prepared Fe_3_O_4_/RGO nanocomposites are easy to obtain with comparable capacitance and stability.

Based on the above discussions, the improved electrochemical properties of as-prepared Fe_3_O_4_/RGO nanocomposites can be ascribed to the following aspects. Firstly, the existence of RGO not only provides plenty of electron transport channels for supercapacitor electrode but also partially accommodates the volume change during cycling. Secondly, the dispersion and intercalation of Fe_3_O_4_ nanoclusters separate RGO sheets and inhibit their restacking, leading to an increased surface area and ensuring the utilization of magnetite. Finally, the unique microstructures of Fe_3_O_4_ can shorten the diffusion path of electrolyte ions and contribute the synergistic effects to RGO. Therefore, the as-prepared Fe_3_O_4_/RGO nanocomposites are considered as prospective candidates for future electrode materials in energy storage.

## 4. Conclusions

In this paper, Fe_3_O_4_/RGO nanocomposites were prepared by coprecipitation in an oxygen-free environment with some additives. Fe_3_O_4_ nanoparticles were dispersed homogeneously with average sizes of 3–8 nm, which were self-assembled as irregular oval-shaped nanoclusters and wrapped by RGO sheets. XRD and XPS analysis confirmed their preferable crystallization and high purity. Compared with pure Fe_3_O_4_, Fe_3_O_4_/RGO nanocomposites deliver improved specific capacitance of 572.8 F g^−1^ at the scan rate of 5 mV s^−1^ and better cycling stability remaining 86.9% after 5500 cycles. The excellent electrochemical properties were ascribed to high electron transport, increased interfaces and positive synergistic effects between Fe_3_O_4_ and RGO, which enlightened an effective strategy in producing supercapacitor electrodes for energy storage devices.

## Figures and Tables

**Table 1:** Comparisons on electrochemical performance of Fe_3_O_4_ and related carbonaceous composites as supercapacitor electrodes in the present study and literature survey.

Materials	Morphology	Specific Capacitance (F g^−^^1^)	Electrolyte	Stability	Ref.
**Fe_3_O_4_**	Hollow microspheres	294 (0.5 A g^−^^1^)	8 M KOH	90.8% after 500 cycles	[9]
**Fe_3_O_4_**	Microflowers	183 (1.0 A g^−^^1^)	0.5 M Na_2_SO_3_	65.0% after 5000 cycles	[10]
**Fe_3_O_4_**	Nanoparticles	383.2 (0.5 A g^−^^1^)	1 M Na_2_SO_3_	83.6% after 2000 cycles	[11]
**Fe_3_O_4_**	Nanoparticles	169.1 (0.5 A g^−^^1^)	1 M KOH	69.2% after 5500 cycles	This work
**Fe_3_O_4_/C**	Nanoparticles/Nanotubes	187.1 (1.0 A g^−1^)	1 M Na_2_SO_3_	80.2% after 1000 cycles	[12]
**Fe_3_O_4_/C**	Nanorods/Nanoparticles	275.9 (0.5 A g^−1^)	1 M Na_2_SO_3_	81.2% after 500 cycles	[13]
**Fe_3_O_4_/RGO**	Nanosheets/Nanosheets	297.0 (4.4 A g^−1^)	2 M KOH	91.4% after 9600 cycles	[14]
**Fe_3_O_4_/RGO**	Nanorods/Nanosheets	315 (5.0 A g^−1^)	1 M KOH	95.0% after 2000 cycles	[15]
**Fe_3_O_4_/RGO**	Nanodiscs/Nanosheets	1149 (1.5 A g^−1^)	6 M KOH	97.5% after 10,000 cycles	[21]
**Fe_3_O_4_/RGO**	Nanoflowers/Nanosheets	454.3 (1.0 A g^−1^)	2 M KOH	94.0% after 10,000 cycles	[26]
**Fe_3_O_4_/RGO**	Nanoparticles/Nanosheets	317.4 (0.5 A g^−^^1^)	1 M KOH	86.9% after 5500 cycles	This work

## Data Availability

The data presented in this study are available from the corresponding authors upon reasonable request.
